# Estimating fine-scale changes in turbulence using the movements of a flapping flier

**DOI:** 10.1098/rsif.2022.0577

**Published:** 2022-11-09

**Authors:** Emmanouil Lempidakis, Andrew N. Ross, Michael Quetting, Baptiste Garde, Martin Wikelski, Emily L. C. Shepard

**Affiliations:** ^1^ Department of Biosciences, Swansea University, Singleton Park, Swansea, UK; ^2^ School of Earth and Environment, University of Leeds, Leeds, UK; ^3^ Max Planck Institute of Animal Behavior, Radolfzell, Germany; ^4^ Centre for the Advanced Study of Collective Behaviour, University of Konstanz, Konstanz, Germany

**Keywords:** remote sensing, gust, ultralight, acceleration, uncrewed aerial vehicle, bio-inspiration

## Abstract

All animals that operate within the atmospheric boundary layer need to respond to aerial turbulence. Yet little is known about how flying animals do this because evaluating turbulence at fine scales (tens to approx. 300 m) is exceedingly difficult. Recently, data from animal-borne sensors have been used to assess wind and updraft strength, providing a new possibility for sensing the physical environment. We tested whether highly resolved changes in altitude and body acceleration measured onboard solo-flying pigeons (as model flapping fliers) can be used as qualitative proxies for turbulence. A range of pressure and acceleration proxies performed well when tested against independent turbulence measurements from a tri-axial anemometer mounted onboard an ultralight flying the same route, with stronger turbulence causing increasing vertical displacement. The best proxy for turbulence also varied with estimates of both convective velocity and wind shear. The approximately linear relationship between most proxies and turbulence levels suggests this approach should be widely applicable, providing insight into how turbulence changes in space and time. Furthermore, pigeons were able to fly in levels of turbulence that were unsafe for the ultralight, paving the way for the study of how freestream turbulence affects the costs and kinematics of animal flight.

## 1. Introduction

The impact of atmospheric turbulence on flying animals represents an important frontier [1–3], as the effects of turbulence on flight energetics and route selection are far less studied than the effects of wind [4,5]. Turbulence is broadly defined as a measure of rapid changes in wind velocity at small scales and is mainly driven by shear forcing or thermal heating, although it can also be generated by warm and cold fronts. Shear forcing results from the interaction of wind with the topography and obstacles on the surface, leading to flow instabilities and eddy production. While shear forcing depends on the presence of mean wind, turbulence produced by thermal heating can occur even in the absence of a mean flow, through convective instability, i.e. when warm air near the surface rises through cooler, denser air. Within the atmospheric boundary layer, and at the usual altitudes at which animals fly, turbulence is influenced by both processes, depending on the strength of the wind and heat fluxes, the altitude above ground, land cover and topography.

Thermal-driven turbulence has a strong upward component, which a range of birds exploit to reduce flight costs through thermal soaring [6,7], particularly in species adapted to soaring flight, where the distribution of thermal updrafts can influence movement paths from local to regional scales [8,9]. Much less is known about the impact of gustiness, which is associated with both thermal- and mechanically driven turbulence. Flying animals will experience gusts when velocity fluctuations are of a magnitude at least as large as the wing chord or span. Experiments, mostly done in laboratory conditions, have shown that gusts can impact flight control [10,11] and flight costs [12–14]. As such, gustiness is relevant for birds irrespective of their flight style and body mass. In fact, gusts should be most relevant for animals flying close to the ground, where an inability to respond to gusts could ultimately result in a collision. Indeed, it has been suggested that these risks explain why gulls soaring above buildings increased their distance to the buildings with increasing wind strength [15].

Despite the potential importance of turbulence in animal movement, documenting spatially and temporally explicit changes in gustiness remains challenging over fine scales (tens to hundreds of metres) [16]. Most weather stations do not report turbulence intensity or vertical wind velocity, and when *in situ* measurements are made, conditions at a single location near the ground are not representative of those at higher altitudes or along an entire flight track. Weather balloons can reach high altitudes in the atmosphere and are useful in providing vertical profiles of atmospheric turbulence and stability (unstable, neutral or stable), but these also offer *in situ* measurements of paths that cannot be controlled but are rather determined by wind speed and direction, as well as being costly [17]. Reanalyses models, which combine global circulation forecast models, observational data and an assimilation scheme to produce estimates of past atmospheric variables [18], provide estimates of vertical velocity and turbulence strength (e.g. the turbulent kinetic energy) with excellent spatial coverage. However, the temporal and spatial resolutions tend to be relatively coarse, at best hourly and of the order of tens of kilometres respectively. Reanalyses models therefore cannot resolve changes in fine-scale gustiness that birds would experience along their flight path [9].

Large eddy simulation models (LES) can also be used to realistically assess the conditions associated with flight (e.g. for modelling convective velocity w* [19]) over a wide range of terrains and conditions. LES can produce fine-scale predictions (down to a few centimetres) by nesting a high resolution model within a lower resolution regional model(s). However, the computational costs tend to constrain their application according to the combination of the study area size, desired temporal/spatial resolution, complexity and available computational resources [20,21].

Remote sensing solutions, namely LiDAR (light detection and ranging), are also capable of measuring near-surface changes in turbulence of the order of seconds and tens of metres, by scanning at different angles to infer the three velocity components. In most cases, turbulence is quantified by the vertical velocity of a vertically pointed LiDAR (e.g. [22]). The critical limitation of LiDAR for ecologists is the availability and expense of such sensing equipment, which means that measurements tend to be made in specific locations in association with meteorological research programmes and often do not have wide spatial coverage depending on the instrument and scanning pattern [23]. LiDAR also incorporates errors depending on atmospheric stability and scanning method, and often assumes that the three-dimensional flow is horizontally homogeneous (an assumption usually not valid, i.e. over complex terrain).

Recently, researchers have shown that the movements of the birds themselves can be used to quantify wind and thermal strength [19,24]. Indeed, large tracking datasets mean that these Lagrangian approaches can be used to quantify conditions over substantial areas [25]. It is clear from flying in aircraft that turbulence can cause fluctuations in altitude and body motion. In fact, the clear air turbulence has been estimated using routine measurements of aircraft motion [26] and the variation in vertical acceleration [27]. Similarly, Laurent *et al.* [2] showed that there was a linear relationship between the body accelerations of a single 5 kg golden eagle (*Aquila chrysaetos*) during gliding flight and the atmospheric turbulence (specifically, the body accelerations exhibited power spectra characteristic of turbulence and that increased in proportion to the turbulence intensity). This demonstrates the potential for using high-frequency data from animal-attached loggers to estimate changing levels of turbulence experienced by animals in flight.

Our aim was to establish whether the highly resolved movements recorded using animal-attached data loggers can be used as qualitative proxies for turbulence in flapping fliers. As this is the predominant type of flight used by birds, the ability to extract information on turbulence from onboard loggers would represent a powerful technique allowing researchers to monitor the aerial environment. On the one hand, flapping fliers tend to be smaller than those that rely on gliding flight, potentially making their movements more sensitive to the movements of the air. But against this, the motion of the wings could compensate for much of the turbulence spectrum, resulting in little displacement of the body from a straight and level course. In order to investigate this, we flew an ultralight (ATOS VRS280, www.a-i-r.de) at the same time and in the same area as homing pigeons (*Columba livia*). The birds and the ultralight were equipped with high-frequency data loggers recording altitude and body acceleration, and the ultralight was also instrumented with a tri-axial anemometer to provide independent estimates of turbulence. We used the resulting data to assess (1) the performance of flight metrics based on variation in pressure (altitude) and body acceleration as turbulence proxies and (2) whether the predictive power of the proxies varied according to whether data were collected onboard the fixed wing aircraft or flapping fliers.

## 2. Material and methods

### 2.1. Data collection

**Figure 1.**
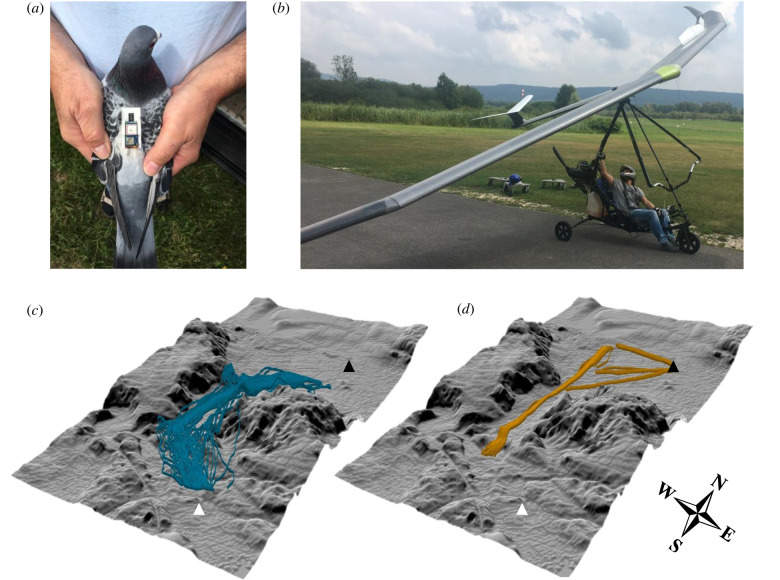
The logging platforms used in this study and corresponding flight tracks. (*a*) The combined logger unit used on the pigeon's back, (*b*) the ultralight, (*c*) pigeon tracks and (*d*) ultralight flight legs. Black and white triangles indicate the location of the release site and the loft, respectively. The parts of the tracks where pigeons performed orientation circles near the release site or loft, and the ultralight flight legs that did not form a straight line, were removed from the analysis, producing pigeon and ultralight flights with mean durations of approximately 293 and approximately 155 s respectively.

Data were collected near Radolfzell in Germany (47°44'42.76″N, 8°57'59.39″E) within an area of 36.6 km^2^ characterized by a narrow valley between two forested hills (figure 1*c,d*), where elevation ranged from 494 to 715 m (electronic supplementary material, figure S1). Flight data were collected over six days in July 2018, eight days in April 2019 and nine days in July 2019. This ensured that we sampled a wide range of convective conditions, as well as wind strengths and directions. Data were also collected during morning and afternoon sessions, as turbulence and wind strength tended to be higher in the afternoons. Wind speed was logged at the pigeon release site every five seconds using a Kestrel 5500 anemometer (Kestrel Instruments, USA) stationed 5 m above ground.

Pigeons were brought from the loft to the release site by car; a straight-line distance of 5.7 km (see Garde *et al.* [28] for details). Both the pigeons and the ultralight were equipped with a ‘Daily Diary’ logger (Wildbyte Technologies, Swansea University, UK), recording tri-axial acceleration (smallest step/resolution 0.065 m*g* per bit) and barometric pressure (using Bosch pressure sensor BMP280 with a relative accuracy of ±0.12 hPa, equivalent to ±1 m), and a GiPSy-5 GPS logger (Technosmart, Rome, Italy). The two tags were connected to the same battery and integrated in a single three-dimensional printed housing [28]. The Daily Diary tag was programmed to take the time from the GPS logger in order to time-synchronize the two datasets. The combined system was attached to the back of ten pigeons using a Velcro patch attached to the down feathers with cyanoacrylate glue (figure 1*a*) [29]. The total unit had an approximate mass of 18 g, accounting for <4.5% of the mean pigeon body mass (455 g). One logger was also attached to the front of the ultralight frame (figure 1*b*). The ultralight had a mass of 173 kg, including the pilot and petrol, and a wingspan of 12 m. Tags were programmed to record barometric pressure at 20 Hz and acceleration at 200 Hz, with the exception of two pigeon flights where measurements were taken at 4 Hz and 40 Hz, respectively. The GPS sampled locations at 1 Hz except for the flights in April 2019, where sampling frequency was 5 Hz. For consistency, these flights were subsampled to 1 Hz.

Prior to our trials pigeons had been flown with dummy loggers from the release site >30 times to remove changes associated with route learning. We released six pigeons on a given day from the same release site, with 11–13 min between each release. Pigeons were released to fly solo so that any turbulence they encountered was the result of environmental conditions and not other birds [30]. Birds were released when the ultralight reached the release site, to ensure that both the bird and the ultralight were flying in similar wind conditions. Once the bird was released, the ultralight flew a straight track from north to south (figure 1*d*), which largely coincided with the main pigeon flight route (figure 1*c*), with the ultralight remaining behind the focal bird at all times. The ultralight maintained a fixed altitude and speed during the flight, before returning to the release site for the next release.

While every attempt was made to maintain the same ultralight path, on three days in July 2019, the pilot flew a triangular path close to the release site and before the valley because of mechanical difficulties and/or unsafe wind conditions. This triangular path was split into two or three sections to extract flight legs that formed a relatively straight line. This reduced the effect of the ultralight's motion on the ultrasonic anemometer measurements (see below). The result of this process was 42 shorter flight legs (approx. 40–60 s long) included in a total of 88 flights where a Daily Diary logger was attached on the ultralight. Forty-two more flights were included in the analysis of turbulence (see below) where only ultrasonic anemometer data were available. These additional flights were only included in the comparison between the estimated turbulence and the pilot's rating of turbulence (see below). Data were collected with approval from the Animal Welfare Ethical Review Body of Swansea University (approval number: IP-1718-23) and the Regierungspräsidium Freiburg (reference number: G-17/92).

### 2.2. Turbulence estimation

A triaxial ultrasonic anemometer (uSonic-3 CLASS A; electronic supplementary material, figure S2) was mounted on the front of the ultralight (2.2 m upstream of the propeller, which remained on during test flights) and programmed to record at 20 Hz. The three wind components were used to estimate the overall turbulence level for each flight leg, and this, in turn, was used to examine the performance of turbulence proxies at scales of a few hundred metres (see Data analysis).

Turbulence intensity can be defined as the wind speed fluctuations relative to the mean wind speed. However, measurements made by the ultrasonic anemometer also included a contribution from the motion of the ultralight itself. Full estimation and subtraction of the ultralight's contribution require high-resolution measurements of the orientation and acceleration of the platform; however, this is a complex process requiring very accurate measurements synchronized with the wind speed data [31–33]. This approach is required for calculating turbulent fluxes. Since our aim was to provide a qualitative measure of changes in turbulence levels, we developed a simpler approach using the anemometer measurements alone.

The log power spectrum density of the root mean square of the three velocity components is predicted to follow a −5/3 slope for homogeneous, isotropic turbulence. This holds over a wide range of frequencies (the whole inertial subrange) as energy cascades from low to high frequencies (larger to smaller scale eddies) until it is dissipated through viscosity [34]. Our spectra were heavily contaminated by the ultralight's motion, especially at lower and higher frequencies. Nonetheless, it should be possible to identify a range of frequencies where contamination from the ultralight motion is small and this slope can be fitted. Visual inspection of the log power plots revealed that frequencies between approximately 10^−2.3^ and 10^0^ (approx. 0.05 to 1 s) were typically the least contaminated in the inertial subrange, and the most appropriate for fitting the ideal −5/3 power law (electronic supplementary material, figure S3). Our method automatically fitted the −5/3 line within this range of frequencies and objectively selected the subrange with the best fit for each ultralight leg (that with the lowest root mean square error), subject to the subrange spanning at least half a decade.

From this fit we obtain the constant of proportionality between the power spectrum density and the frequency to the power of −5/3. This constant of proportionality is directly related to the eddy dissipation rate, which is equal to the rate of turbulence production at large scales (assuming the turbulence is statistically stationary), and hence provides a measure of turbulence. This follows because energy in the inertial subrange is not generated or lost, but rather cascades from larger to smaller scales. Indeed, this proportionality is often used in meteorology to estimate the dissipation rate from ultrasonic anemometers (e.g. [35]), and similar approaches have been used to measure clear air turbulence using the eddy dissipation rate from aircraft [26,27]. We, therefore, took the proportionality of the fit for the power law relationship (between the power and the frequency^(−5/3)^ as a measure of the turbulent energy. We also compared turbulence estimates from the ultralight anemometer with a qualitative assessment of turbulence made by the pilot on a scale of 0–5 (0: no turbulence; 5: highest turbulence). This is analogous to the turbulence observations that are routinely provided verbally by pilots in the form of pilot reports (PIREPs). Here, the turbulence level is determined by a pilot's subjective experience of the aircraft response to turbulence [27]. Computations of our qualitative measure of turbulence strength were conducted in Python v. 2.7.15 [36].

### 2.3. Data analysis

Estimates of turbulence from the anemometer on the ultralight were used to assess the performance of potential turbulence proxies based on pressure and acceleration measurements on both flying bodies. We predicted that turbulence would cause vertical displacements from a level course in flying bodies that should be evident in the barometric pressure data (noting that the pressure sensor was mounted parallel to the flow and the top was covered by the printed tag housing to minimize the influence of airspeed on the signal). To assess this we smoothed the pressure values over two seconds and calculated the mean pressure difference per second. We also calculated the pressure fluctuations by subtracting pressure smoothed over 30 s (after testing different windows) from the raw values.

We also tested the performance of acceleration-based proxies, which could have advantages over pressure-based metrics because they can capture lateral, as well as vertical, displacements from a straight course. For this we used the vectorial static body acceleration (VeSBA), calculated as the root mean square of the three smoothed acceleration channels, using a two-second smoothing window [37]. Smoothing is a simple, but commonly used, way of removing much of the high frequency ‘dynamic’ component of the acceleration and isolating the ‘static’ or gravitational component. VeSBA should equal 1.0 *g* for a body flying straight and level and maintaining a constant velocity. Departures from 1 *g* can occur when the flying body is acted on by an external unbalanced force, such as turbulence, which can cause (primarily) vertical and lateral displacements. We, therefore, predicted that increasing turbulence would result in greater displacements from a straight and level flight path, producing a positive correlation between VeSBA and turbulence strength. We calculated the fluctuations in VeSBA by subtracting 1.0 *g* from VeSBA values.

In order to account for the difference in resolution between turbulence (one estimate per flight leg), and VeSBA and pressure (sub-second resolution), we calculated four metrics for each quantity per flight leg, the interquartile range (IQR), median, variance and the sum of all absolute values normalized by the flight leg's sample size (SUM), as a measure of the area under the curve (AOC). The SUM and the median were used to identify the magnitude of the fluctuations in pressure and VeSBA, and the IQR and the variance indicated the level of variability per flight (i.e. how variable the mean pressure difference was per second). This process resulted in a set of 18 metrics.

The performance of all metrics in predicting turbulence strength was evaluated with generalized additive effect models with Gaussian errors (GAM, package ‘mgcv’ v. 1.8.31 [38]). Turbulence measured by the ultrasonic anemometer was included as the response, and one flight metric based on either barometric pressure or VeSBA as the predictor in each case. This allowed us to test for linear and nonlinear relationships across the large set of models. Each ultralight flight leg (*n* = 88) was associated with the turbulence measured within it, while each pigeon flight (*n* = 66) was assigned the turbulence estimate of the closest ultralight flight in time (range: 0.2–107 min), with closest ultralight flight for two pigeon flights being greater than one hour (excluding these flights did not significantly change the results). We assessed the influence of the time delay between the pigeon flight and the associated ultralight flight by including this in the model as a fixed effect. We also assessed the effect of flight altitude in the pigeon models by including the difference in median altitude between a pigeon flight and its closest ultralight flight in time. One model was run for each single metric and models were ranked by their predictive ability in terms of Akaike's information criterion (AIC).

The potential effect of different tags or pigeons was assessed by running the same models as generalized additive mixed effect models (GAMMs) with the addition of tag ID or pigeon ID (in the case of the pigeon models) as a random effect (intercept). All models included the date and the mean coordinates of each flight, using the corARMA and corSpatial functions (nlme package v. 3.1.148 [39]) to account for temporal and spatial autocorrelation respectively. The final models were evaluated for outliers, uniformity, over-/under-dispersion and spatial/temporal autocorrelation using the DHARMa package v. 0.3.3.0 [40]. All statistical analysis was conducted in RStudio v. 1.2.5 [41] and the R programming language v. 3.6 [42].

As an additional ground-truthing step we assessed whether our best turbulence proxies were correlated with estimates of the convective velocity (w*) and the shear velocity (u*), using data from the global reanalysis ERA5 [43] (see electronic supplementary material for the estimation of convective and shear velocities).

## 3. Results

In several cases, birds did not return to the loft immediately but waited until other flockmate(s) were released, before flying back together. These flights, as well as others with incomplete or erroneous data were excluded. Overall, 66 pigeon flights were used to estimate turbulence proxies after excluding the non-solo flights, flights where the barometric pressure sensor recorded unrealistic estimates, flights for days which the ultralight pilot did not fly because of mechanical problems or because of unsafe conditions in the valley and finally flights that included landing breaks (identified in the acceleration signal). A total of 88 ultralight flights were used to estimate turbulence proxies after excluding flights for which the ultrasonic anemometer did not record data because of malfunction or low battery and flights without a daily diary logger onboard. Pigeons flew slightly lower than the ultralight with mean flight heights of 81 m (s.d. ± 24 m) and 116 m AGL (s.d. ± 13.4 m), respectively.

Our method of fitting the −5/3 power law line to the turbulence spectrum from the anemometer gave linear fitting errors >0.2 (root mean square) for 24% of the ultralight flights. This suggests that (i) the mean flow and/or strength of the turbulence changed over the length of the flight leg and/or (ii) the part of the power spectra selected for analysis still contained some contamination from the motion of the ultralight. Nonetheless, there was a positive relationship between the ultrasonic anemometer estimates of turbulence and the pilot's turbulence score on a given day (Pearson correlation coefficient = 0.73, *t* = 12.11, d.f. = 128, *p*-value < 2.2 × 10^−16^) (linear fit: adj. *R*^2^ = 0.53, *n* = 130; electronic supplementary material, figure S4).

### 3.1. Performance of turbulence proxies (i) pigeon flight data

A positive linear/near-linear relationship was identified between the anemometer estimates of turbulence and the pigeon-based proxies in almost all of the top models in terms of AIC (table 1; electronic supplementary material, figure S5), which included both pressure- and VeSBA-based proxies. The top two models were proxies of pressure fluctuations, while three models of mean pressure difference per second (SUM, IQR and variance) followed in the top 10 models. Pressure proxies ranked higher than VeSBA proxies and had the highest *R*^2^, with only three proxies based on VeSBA included in the top 10 models. Overall, AIC scores differed between models and the adj. *R*^2^ values ranged between 0.21 and 0.41. Neither tag ID nor pigeon ID was significant. The time delay between the pigeon and ultralight flights was significant in only two of the top 10 models and it did not improve the *R*^2^ values substantially. The difference in flight altitude between the pigeon and ultralight was significant in five of the top 10 models (electronic supplementary material, table S1), with turbulence increasing as pigeons flew higher than the ultralight. Given that most studies will not have turbulence information from an independent platform flying near their study animal, we report model parameters from models without time delay or difference in altitude.

**Table 1.** Top 10 pigeon proxy models ranked by AIC. ‘IQR’, ‘VAR’, ‘MED’, ‘SUM’ are used to indicate that the model is the interquartile range, variance, median and area under the curve of each property. The estimated degrees of freedom (EDF) indicate the extent to which relationships are linear, with values closer to 1 describing a linear fit and closer to 2 curvilinear.

model	*p*-value	EDF	adj. *R*^2^	dev. expl.	AIC	ΔAIC
raw pressure fluctuations_ IQR	<0.001	1.57	0.41	0.43	176.59	—
raw pressure fluctuations_SUM	<0.001	1.13	0.40	0.41	177.33	0.74
raw pressure fluctuations_ MED	<0.001	1.20	0.38	0.39	179.58	2.99
raw pressure fluctuations_VAR	<0.001	1.71	0.34	0.36	184.81	8.21
mean pressure difference per s_SUM	<0.001	1.70	0.30	0.32	188.26	11.67
mean pressure difference per s_IQR	<0.001	1.80	0.29	0.31	189.01	12.42
mean pressure difference per s_VAR	<0.001	1.80	0.27	0.29	191.31	14.72
VeSBA fluctuations_VAR	<0.001	1.75	0.27	0.29	191.31	14.72
VeSBA_VAR	<0.001	1.40	0.22	0.24	195.28	18.69
VeSBA fluctuations_SUM	<0.001	1.00	0.21	0.22	195.65	19.06

### 3.2. Performance of turbulence proxies (ii) ultralight flight data

Proxies from metrics recorded on the ultralight performed less well than metrics from pigeon movements, with the *R*^2^ from the top model being 0.33 (electronic supplementary material, table S2), compared to a maximum of 0.41 in the pigeon flight models. Nonetheless, similar to the pigeon proxies, models of pressure ranked higher than models of VeSBA. As with the pigeon models, all pressure summary statistics were included in the top 10 models. However, models of mean pressure difference per second ranked higher than models of pressure fluctuations, with the model that ranked first in the pigeon models, ranking seventh place in the ultralight models. Tag ID was significant, which was unsurprising as the same tag was used for all flight legs of the same day, and a small number of tags were used on the ultralight compared to the pigeons.

**Figure 2.**
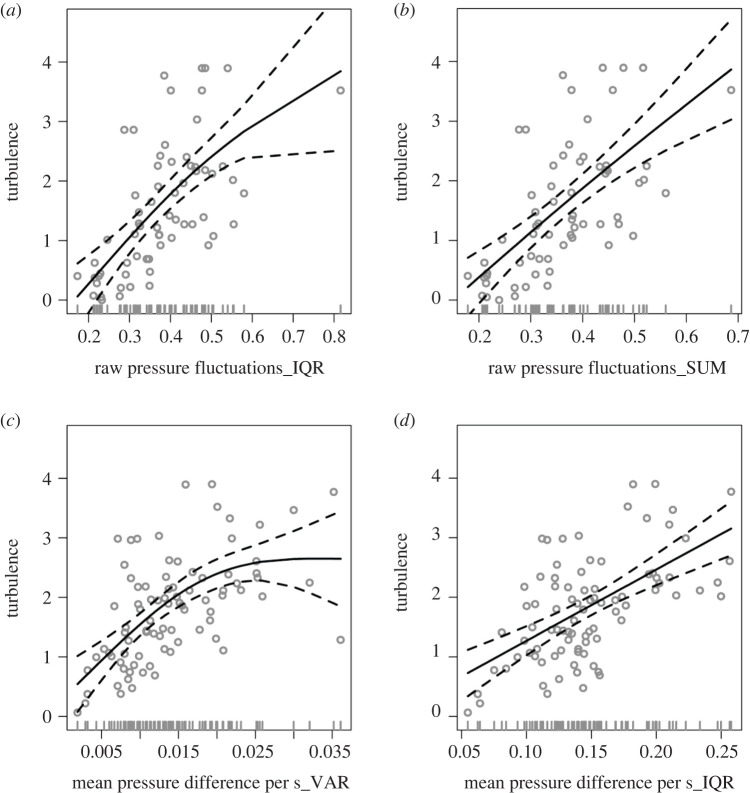
Turbulence predicted from the top two models based on pigeon movement metrics, the interquartile range of pressure fluctuations (*a*) and the sum of all absolute values of pressure as a measure of the area under the curve (*b*), and from the top two models based on ultralight movement metrics, the variance (*c*) and interquartile range (*d*) of the mean difference in pressure per second, per flight. The top two models based on pigeon flight had an adjusted *R*^2^ of approximately 0.4 and the two ultralight based models an *R*^2^ of 0.33 and 0.31, respectively. Black solid curves indicate the predicted fit (and dashed lines the 95% confidence intervals) and grey points the raw observations.

Near linear relationships were identified in the top 2 of the 10 best models for pigeon- and ultralight-based proxies (figure 2). While the majority of pigeon-based proxies had an approximately linear relationship with turbulence this varied between the pigeon and ultralight proxies, with more nonlinear relationships predicted for the ultralight, although many of these nonlinear relationships were strongly influenced by very high/low proxy values where there were few data points (electronic supplementary material, figures S5 and S6).

### 3.3. Comparing pigeon flight turbulence proxies with thermal and mechanical turbulence

**Figure 3.**
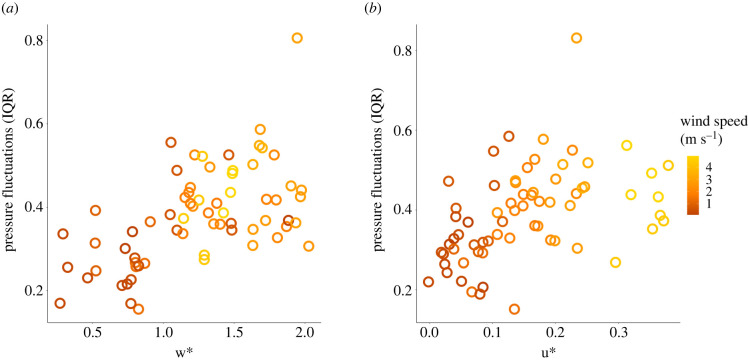
Comparison of the best pigeon turbulence proxy (the interquartile range of pressure fluctuations per flight) with (*a*) thermal uplift (w*) and (*b*) shear velocity (u*) as estimated using the ERA5 global reanalysis. Each point represents the interquartile range of each flight with colour indicating the mean wind speed during each flight (*n* = 66 flights).

The best pigeon proxy in terms of both *R*^2^ and AIC (raw pressure fluctuations IQR) was positively correlated with both the hourly convective velocity (w*) and shear velocity (u*) (Spearman correlation coefficients: 0.58, *S* = 2104.96, *p*-value = 2.7 × 10^−07^ and 0.59, *S* = 20 534.33, *p*-value = 1.5 × 10^−07^, for w* and u*, respectively). The latter, in turn, increased with the ERA5 wind speed (figure 3).

**Figure 4.**
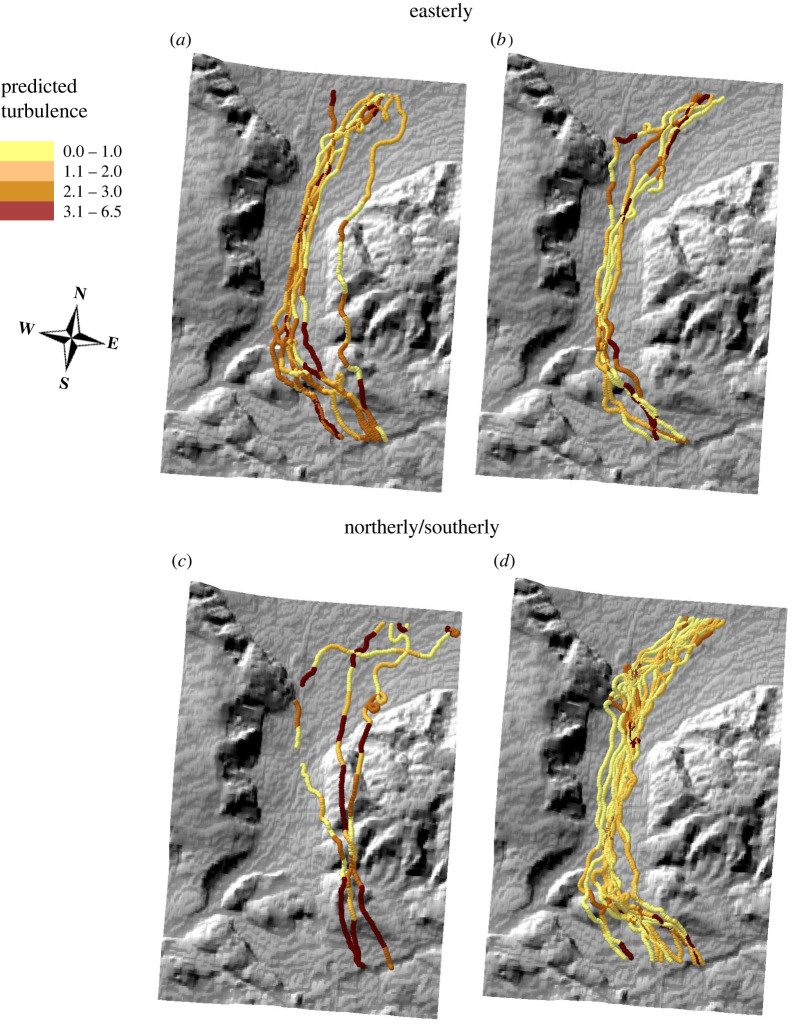
Spatial variation in turbulence as predicted from the fluctuations in pressure recorded onboard pigeons (specifically the interquartile range). Predictions were made for (*a*) highly turbulent conditions (mean ≥ 3.0 per flight, mean wind speed = 2.9 m s^−1^, *n* = 7 flights) and (*b*) low turbulence (mean ≤ 1.5 per flight, mean wind speed = 1.3 m s^−1^, *n* = 5 flights), both in easterly winds, and (*c*) high turbulence (mean wind speed = 2.7 m s^−1^, *n* = 3 flights) and (*d*) low turbulence (mean wind speed = 1.7 m s^−1^, *n* = 12 flights), in northerly or southerly winds.

Mapping the turbulence using the same proxy summarized over 15-second sections of the flight path revealed spatial patterns in turbulence (figure 4) that also varied with wind direction. Predicted turbulence was particularly high in the inlet and the outlet of the valley in easterly winds (figure 4*a*,*b*), whether or not overall turbulence was high. This pattern was also seen in the measured turbulence and corroborated by the pilot's experience. Nonetheless, the valley inlet and outlet were not particularly turbulent in northerly or southerly winds (figure 4*d*) when turbulence conditions were weak. Furthermore, birds appeared to avoid the southerly end of the valley in northerly/southerly winds when the mean turbulence was high (figure 4*c*).

## 4. Discussion

Previous studies have demonstrated that animal movement can be used to quantify the strength of thermal updrafts [9,19,44] and determine the wind vector [24], at scales and in locations that are not possible using traditional meteorological approaches. Here we show that the highly resolved, vertical displacements of a flapping flier can provide qualitative predictions of turbulence at the scale of a few hundred metres. Our proxies are similar in concept to those developed for aviation [27], demonstrating the widespread utility of simple measurements derived from onboard accelerometers and pressure sensors. Furthermore, the fact that turbulence generally followed an approximately linear relationship with the proxies (particularly for birds but also in some cases for the ultralight proxies) means that relative changes in turbulence should be straightforward to approximate. This simple relationship is in line with previous work that identified a linear correlation between the turbulence spectrum and the spectral composition of acceleration recorded onboard a golden eagle in gliding flight [2].

Turbulence appeared to cause greater displacements for pigeons in flight than for the ultralight, as proxies derived from the ultralight flight did not perform quite as well. This will primarily be due to the substantial differences in mass and wing loading between the two systems, with a given eddy accelerating the larger ultralight to a lesser extent. Mean airspeeds of the ultralight and pigeon were similar (21.5 and 19.9 m s^−1^, respectively), but the objectives are also likely to have differed, as the pilot aimed to maintain a constant airspeed and straight and level flight, therefore compensating for turbulence along the flight path. It is likely that the pilot's ability to achieve this varied with the scale of the eddies, which may also have affected the performance of pressure and VeSBA proxies from the ultralight. This, combined with the contamination of the three velocity components from the aircraft's motion, suggests that the pigeon-based proxies may even provide a better representation of turbulence variation than the ground-truth ultrasonic anemometer measurements. Nonetheless, the good agreement between turbulence estimates from the anemometer measurements, the pilot's scores, reanalysis data and proxy values gives confidence in the performance of our proxies.

Pressure proxies performed better than VeSBA proxies for both the pigeon and ultralight data, despite the fact that the variability in both proxies was positively correlated. In fact, the seven best pigeon proxies in terms of AIC were models of pressure, suggesting that turbulence caused more pronounced vertical than lateral displacements in these birds (as pressure-based proxies relate to changes in the vertical axis whereas VeSBA will be sensitive to both vertical and lateral displacements).

While we have not tested how pigeons respond to turbulence in terms of their kinematics and speed selection, it is clear that turbulence causes substantial variability in their vertical movements over fine scales, and that this increases with turbulence strength. The motion of the pigeons' wings, therefore, did not dampen out all the turbulence that the birds encountered. Variability in the pigeon trajectories has previously been interpreted as protean behaviour, with it conferring potential benefits as a predator avoidance strategy for solo-flying birds [28]. These explanations are not necessarily mutually exclusive. The study by Garde *et al.* [28] used a subset of our data (29 of 66 flights) that were performed under relatively weak turbulence levels, with mean and maximum values of 0.9 and 2.5, compared to 1.6 and 4.7 in the current study. They also reported a lower variance in climb rate (mean and maximum 0.9 and 2.0 m s^−1^ per flight, compared to 1.6 and 6.7 m s^−1^ reported here). Therefore, while some of the variability in pigeon flight behaviour [28] is due to turbulence, there may still be a baseline level of variability in birds flying solo and in low turbulence that could represent an anti-predator response. In both cases, what remains unknown is the power costs associated with variation in the flight trajectory.

The behaviour of the birds themselves, including the route selection, can provide insight into the likely costs or benefits of turbulence. Mapping changes in our pigeon-based proxies (estimated over 15 s) along the flight paths revealed patterns in turbulence that varied in both space and time. The high levels of turbulence at the inlet and the outlet of the valley in strong easterly winds are likely the result of wind shear, with the valley itself being more sheltered by the forested hill to the east. While predicted turbulence was lower overall in weaker winds, the strong turbulent features at the inlet and the outlet of the valley generally persisted, which might help explain why birds tended to fly the (lower turbulence) valley route. High turbulence was also predicted over the forested hill when wind direction changed to northerly. This is consistent with wind travelling over an extended level terrain before the mouth of the valley where it is accelerated by the hill with high turbulence over the hill itself (although sample size is small in these conditions). These results also agree with the spatial variability in turbulence strength experienced by the pilot.

Overall, our results demonstrate the capacity of simple, animal-based proxies to estimate fine-scale changes in turbulence over complex terrain. While the absolute values of our proxies may provide a useful guide for other flapping fliers, it is likely that the relationship to turbulence levels will vary with factors including (i) animal mass, airspeed and flight height, (ii) the sampling frequency and tag location, in the case of VeSBA, and (iii) the scale and variation of the turbulence experienced by the animals. A valuable first step in other systems would therefore be to examine the relationship between turbulence proxies and independent environmental data (such as those from ERA5, used in this study), even though those data have coarser spatial and temporal resolution.

Widespread use of tracking technology means that pressure and acceleration data are increasingly available from animals instrumented with data loggers. Our study highlights that these can provide meteorological insight that is logistically difficult and costly to acquire from traditional platforms such as aircraft. Uncrewed aerial vehicles (UAVs) can also provide tractable platforms for the estimation of turbulence [19], particularly in regions where birds do not fly. Nonetheless, planes and UAVs have their own limitations. In our study, there were two days when the turbulence and wind made it unsafe for the pilot to fly the whole route. It is therefore notable that pigeons flew under these conditions, demonstrating that birds can reach areas that are inaccessible to light aircraft (the limitations will be different for UAVs). In some ways this is analogous to the case where seal-borne sensors are used to measure temperature and salinity below the ice, providing invaluable information on oceanographic processes in locations where ship-based studies are infeasible and satellite access is restricted [45,46]. However, our specific case raises intriguing questions about the behavioural, biomechanical [10] and navigational capacities of birds, and other flying animals, that enable them to negotiate an aspect of the flight environment that remains dangerous for aircraft.

## Data Availability

All data that are essential for the findings of this study are available from the Dryad Digital Repository: https://doi.org/10.5061/dryad.w9ghx3fsb [47]. Supplementary material is available online [48].
